# Fusing N-heteroacene analogues into one “kinked” molecule with slipped two-dimensional ladder-like packing†
†Electronic supplementary information (ESI) available: Details of experimental procedure, high-resolution mass spectrometry, ^1^H NMR, ^13^C NMR, computational methodology. CCDC 1420681. For ESI and crystallographic data in CIF or other electronic format see DOI: 10.1039/c5sc03604f


**DOI:** 10.1039/c5sc03604f

**Published:** 2015-11-10

**Authors:** Jing Zhang, Chengyuan Wang, Guankui Long, Naoki Aratani, Hiroko Yamada, Qichun Zhang

**Affiliations:** a School of Materials Science and Engineering , Nanyang Technological University , Singapore , 639798 , Singapore . Email: qczhang@ntu.edu.sg; b Graduate School of Materials Science , Nara Institute of Science and Technology , Ikoma , 630-0192 , Japan; c Division of Chemistry and Biological Chemistry , School of Physical and Mathematical Sciences , Nanyang Technological University , Singapore , 637371 , Singapore

## Abstract

An unexpected “kinked” N-heteroacene with a slipped two-dimensional ladder-like packing feature is produced from the conventional condensation reaction. The as-obtained compound [2,2′]bi(5,12-bis(TIPS)piperazin-3-one[2,3-*b*]phenazine) (**2BPP**) consists of two identical backbones (5,12-bis(TIPS)piperazin-3-one[2,3-*b*]phenazine), which are fused together through a C=C double bond and two intramolecular H-bonds. A study on the charge carrier transport indicates that a **2BPP** single crystal has a hole mobility up to 0.3 cm^2^ V^–1^ s^–1^, while theoretical calculations suggest that this compound might possess potential well-balanced ambipolar charge-transport characteristics.

## Abstract 2

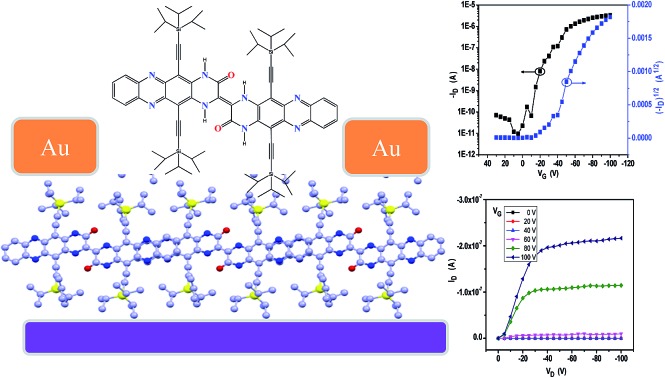
An unexpected N-heteroacene with a slipped two-dimensional ladder-like packing feature shows a hole mobility up to 0.3 cm^2^ V^–1^ s^–1^, while theoretical calculations suggest that this compound possesses potential well-balanced ambipolar charge-transport characteristics.

In past decades, polycyclic aromatic hydrocarbons (PAHs) and N-heteroacene derivatives have attracted a lot of attention, being widely investigated for applications in organic field-effect transistors (OFETs), organic resistance memories (ORMs), organic light-emitting diodes (OLEDs) and organic photovoltaics (OPVs).^1^ Pentacene and its soluble analogue 6,13-bis(triisopropylsilylethynyl)pentacene (TIPS-pentacene) are two of the most popular small-molecule organic semiconductors, exhibiting superior hole transporting mobilities (>1 cm^2^ V^–1^ s^–1^) due to their close intermolecular packing, which is supposed to favor charge carrier transport.^2^ Thus, tailoring one molecular structure to achieve a larger π-conjugated system or a more condense packing mode is highly desirable. Longer acenes are presumed to possess enhanced intermolecular π–π overlap in the solid state, which could lead to high charge carrier mobilities.^3^ However, the instability of large acenes strongly hampers their application in organic electronics. To address this issue, two-dimensional (2D) acenes with both more sextets and sufficient stability are proposed. 2D acenes and their derivatives have large, planar π surfaces, which can provide the increased intermolecular surface overlapping and effectively increase electron delocalization, potentially resulting in enhanced charge carrier transporting properties. For example, single-crystal OFETs of a bistetracene derivative reveal a higher hole mobility up to 6.1 cm^2^ V^–1^ s^–1^ with a remarkable *I*_on_/*I*_off_ ratio of 10^7^.^4^ N-heteroacenes have also been investigated and show p-type, ambipolar or n-type OFET behaviours as the number and position of N atoms in the backbone varies.^5^ OFETs based on a vacuum-deposited *N*-substituted TIPS-pentacene analogue show electron mobilities in the range of 1.0–3.3 cm^2^ V^–1^ s^–1^ under vacuum and 0.3–0.5 cm^2^ V^–1^ s^–1^ in ambient air. To date, most of the explored N-heteroacenes are linearly-fused systems, which can be prepared through the condensation reaction between *ortho*-diamine based acenes and *ortho*-diketone, *ortho*-dihydroxy, *ortho*-dicyano, or *ortho*-dihalogen substituted acenes (Scheme 1).^6^ However, in such synthetic conditions, N-heteroacenes with unusual shapes are rarely discovered, not to mention studied for their applications. In this report, we present an unexpected “kinked” N-heteroacene with a ladder-like packing feature, which was prepared from the conventional condensation reaction. Its new zigzag structure, physical properties and charge transport capabilities have been carefully investigated.

**Scheme 1:**
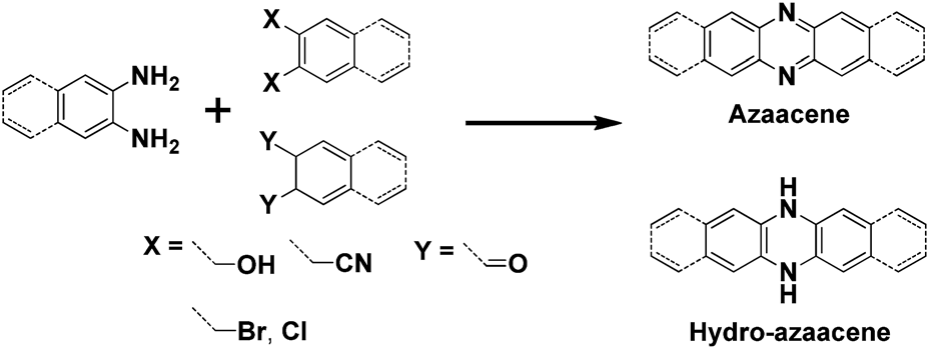
Representative synthetic route to linearly fused N-heteroacenes.

In our previous study, we have already reported that linearly-fused N-heteroacene **THNQ** (shown in Scheme 2) can be prepared through the condensation reaction between 1,4-bis(TIPS)-2,3-diaminophenazine (**BTDP**) and hexaketocyclohexane (**HKCH**), in which we believe that the steric effect of the TIPS group plays a crucial role.^7^ Continuing in this direction, we further studied the reactions between the smaller 1,4-bis(TIPS)-acene-2,3-diamines and **HKCH**.^8^ Surprisingly, when the short 1,4-bis(TIPS)-diaminonaphthalene was used as the starting material, both the star-shaped compound (all the carbonyl groups substituted by N atoms in a yield of 26%) and the linearly-fused product (yield of 17%) were obtained. These results suggest that there might have been other factors besides the steric effect, which could also affect the condensation reaction between **BTDP** and **HKCH**. In this situation, we might miss some important unknown N-heteroacene products. Thus, we reinvestigated this type of reaction and discovered a meaningfully “kinked” compound [2,2′]bi(5,12-bis(TIPS)piperazin-3-one[2,3-*b*]phenazine) (**2BPP**). To the best of our knowledge, this is the first report of the generation of a meaningfully “kinked” N-heteroacene through the conventional substitution reaction.

**Scheme 2:**
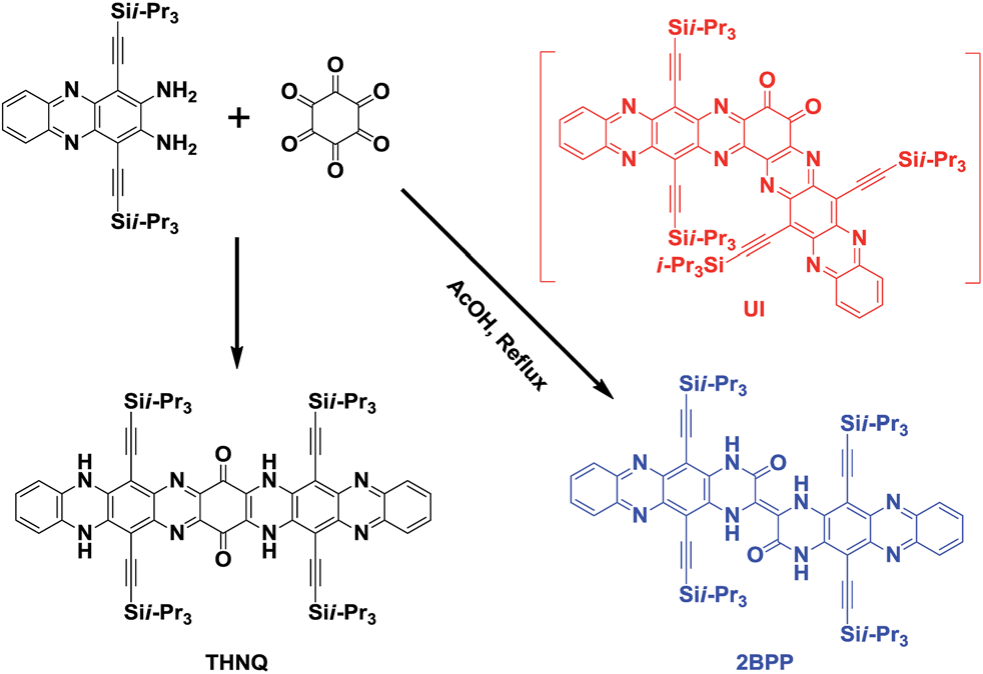
Synthetic route to **2BPP**.

As shown in Scheme 2, the formation of **2BPP** might undergo an unstable intermediate (**UI**) step, in which the diketone could be eliminated instead of participating in the further condensation reaction with the amine groups. The as-formed intermediate could be converted into the final product **2BPP** through a rotation and radical pathway (initiated by light). The possible mechanism has been provided in the ESI (Scheme S1†). Note that the proposed mechanism is different from the previously reported mechanism to eliminate the diketone.^9^ The novel **2BPP** was obtained in a low yield of 2.3% and was fully characterized by high-resolution mass (HR-MS) spectrometry, ^1^H NMR and ^13^C NMR spectroscopy, and single-crystal analysis. It is noteworthy that two 5,12-bis(TIPS)piperazin-3-one[2,3-*b*]phenazine moieties are linked together by one double bond, which makes **2BPP** fully π-conjugated along the backbone. In addition, the expected H-bonds along both sides were assumed to stabilize the large heteroacene system.

Single crystals (CCDC: 1420681 †) of **2BPP** suitable for single-crystal X-ray diffraction analysis were obtained by diffusing the poor solvent acetonitrile into a toluene solution. **2BPP** crystallizes in a triclinic unit cell, space group *P*1(2). The molecular structure of **2BPP** is shown in Fig. 1a. The length of the bond between the C2 and C2′ atoms is 1.35 Å, which is identical to the length of a common C=C bond (1.35 Å), suggesting that the two (5,12-bis(TIPS)piperazin-3-one[2,3-*b*]phenazine, **BPP**) moieties are connected by a C2=C2′ bond. The distance between H1 and O1 or H1′ and O1′ is 1.95 Å, indicating the existence of an intramolecular H-bond. Clearly, the formation of the intramolecular H-bonds with a six-member ring configuration is helpful for stabilizing the planar molecular shape and supports the electron delocalization. In fact, the as-prepared molecule possesses a good planarity from the side view (Fig. 1b). Only a slight twist of **2BPP** can be observed, and the dihedral angle between the **BPP** moiety and the C2=C2′ bond is ∼2.42°. As shown in Fig. 1c, **2BPP** exhibits a slipped 2-D π-stacking motif, similar to that observed for some soluble TIPS-pentacene derivatives. The interplanar distance in **2BPP** is ∼3.27 Å along the *b* axis, less than that of the typical distance for van der Waals interactions, while the centre-to-centre distance between two adjacent molecules is ∼14.65 Å. The slipping angle of two adjacent π-conjugated **BPP**s is ∼45°, contributing to a significant molecular overlap and ensuring the strong π–π interaction. While viewing along the *a* axis, the distance between two adjacent molecules is ∼3.33 Å and in this stacking mode, the characterized centre-to-centre distance is about 17.61 Å with a much smaller slipping angle (∼30°) between two interactional individual **BPP**s leading to poorer electronic coupling. **2BPP** molecules interact with each other to form two-layer **BPP** units. The face-to-face stacking mode results in overlapping between the second layer units and the first layer **BPP**s (Fig. 1d).

**Fig. 1:**
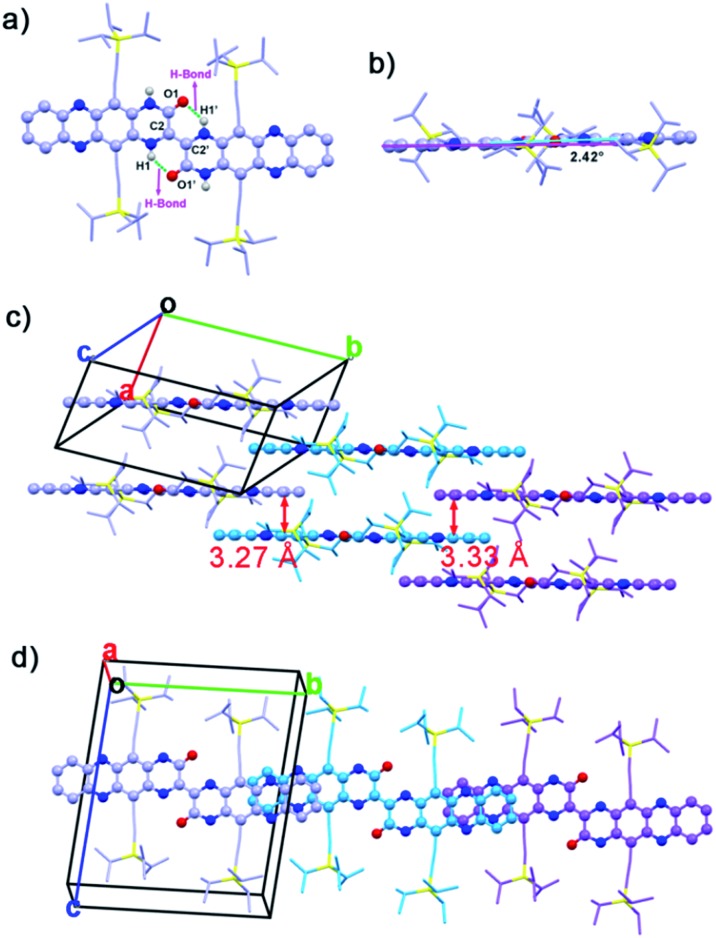
(a) Top & (b) side views of the **2BPP** molecular structure (green dash lines represent the intramolecular H-bond). (c) Interlayer distances between the neighbouring molecules. (d) View of molecular stacking along the *a* axis.

Fig. 2a is the UV-vis absorption spectrum of **2BPP** in CH_2_Cl_2_. **2BPP** has two maximum absorption bands with the maximum absorption at 627 nm and 685 nm. The onset of absorption is 725 nm, from which the optical bandgap (*E*_g_) can be determined to be 1.71 eV. The strong absorption from 650 nm to 725 nm is similar to that of other hydro-azaacenes, and probably comes from the intramolecular charge transfer. The electrochemical properties of **2BPP** were investigated by cyclic voltammetry (CV, Fig. 2b). Unlike other hydro-azaacenes, **2BPP** shows no obvious oxidative peaks. One irreversible reductive peak can be observed with an onset potential (*vs.* Fc^+^/Fc) of –1.29 V, which determines the LUMO level of **2BPP** to be –3.51 eV. The HOMO level was calculated to be –5.22 eV from the LUMO level and *E*_g_. The geometry structure of **2BPP** was optimized by DFT calculations (B3LYP/6-31G*),^10^ and a following frequency analysis was performed to ensure that the optimized structures were the stable states. As shown in Fig. S3,† the LUMO coefficient delocalizes on the whole conjugated backbone, while the HOMO coefficient mainly distributes on the middle electron-rich area. Table 1 summarizes the experimental and theoretical calculated energy levels of **2BPP**. In both the experimental and theoretical calculated results, **2BPP** has relatively high HOMO levels (–5.22 eV for the experimental, and –5.02 eV for the calculated result) and a moderate band gap, which indicates **2BPP** could be used as a promising suitable semiconducting material.

**Fig. 2:**
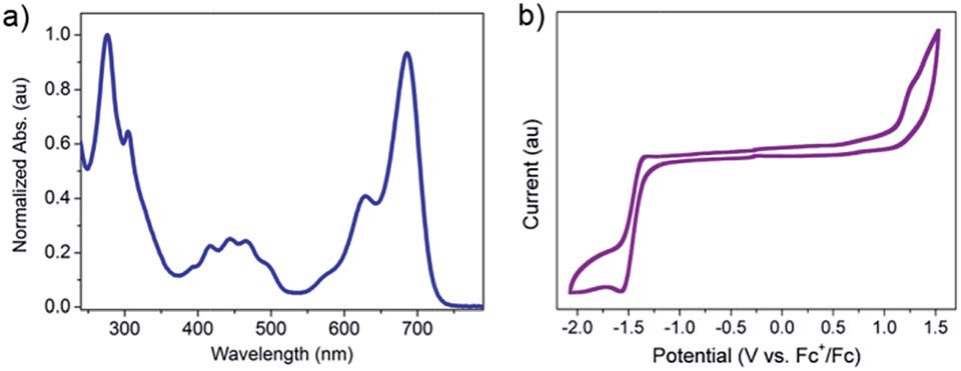
(a) Normalized UV-vis absorption spectrum of **2BPP** in CH_2_Cl_2_. (b) Cyclic voltammetric (CV) curve of **2BPP** in anhydrous CH_2_Cl_2_.

**Table 1:** Summarized energy levels of **2BPP**

Value	HOMO (eV)	LUMO (eV)	*E*_g_ (eV)
Experimental	–5.22	–3.51	1.71
Calculated	–5.02	–2.99	2.04

Crystalline ribbons of **2BPP** were grown on an octadecyltrichlorosilane (OTS)-treated SiO_2_/Si substrate by a drop-casting method. OTS was used to form a siloxane self-assembled monolayer (SAM) on the SiO_2_ layer, which could promote and facilitate molecular self-organization during the growth of ribbons. The optical and AFM images in Fig. 3a and b show that the **2BPP** ribbons display a rhombic shape with unambiguous boundaries, indicating the high quality of the ribbons. The as-obtained micro/nanoribbons are several to tens of micrometers in width, and several tens to hundreds of micrometers in length. Three sharp and strong diffraction peaks at 2*θ* = 5.19, 10.32, and 15.45 degrees are observed in the out of plane X-ray diffraction patterns (XRD) (Fig. 3c), which could be assigned as the (001), (002) and (003) planes, respectively, according to the information from the single-crystal structure. All three peaks in the pattern are well indexed along the (001) lattice plane, indicating that the ribbon-like crystals have high crystallinity. The spacing distance of the (001) plane is 17.13 Å, which is in agreement with the lattice parameters of the *c*-axis (17.27 Å), meaning that the molecules stand up along the *c*-axis with an angle of 73° on the substrate. The major driving force for the self-assembly of the π-conjugated material is the π–π interaction from the adjacent molecules, which causes the superior growth direction along either the *a*-axis or *b*-axis. The measured angle of the lamellar crystal is 100.4°, which is consistent with the 100.2° dihedral angle between the (100) and (010) planes in the crystal structure. From the crystal morphology, it can be clearly seen that the primary growth direction is along the π-stacking direction (*b*-axis). In this direction, the slipping angle between the adjacent overlapped **2BPP** units and the close contact between the adjacent π-scaffolds contributes to the significant molecular overlap and ensures the effective charge transport in the π–π stacking (Fig. 3d). In addition, the intermolecular H-bond interactions are also found in the **2BPP** molecular structure, which would support charge tunneling quickly from the first half to the second half. The π–π stacking direction was parallel to the substrate surface according to the single crystal structure, which makes us believe that this high ordered stacking mode, with a feasible π-electron pathway, favors effective charge transport.

**Fig. 3:**
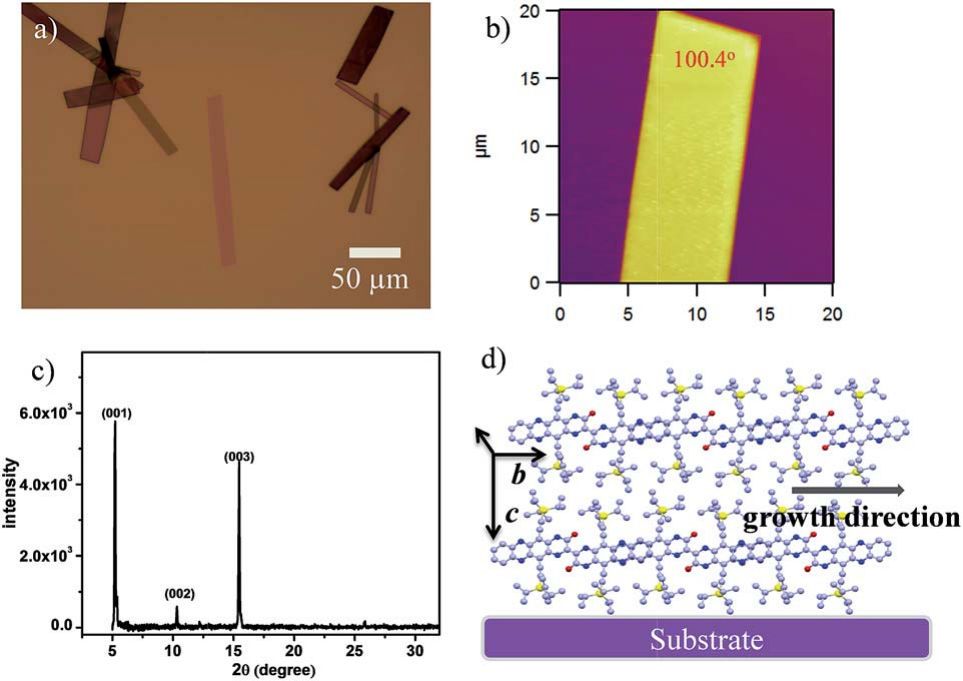
(a) Optical and (b) AFM images of the self-assembled micro/nanoribbons obtained by a solution process. (c) XRD spectrum of the as-obtained micro/nanoribbons. (d) The proposed packing mode of **2BPP** in the ribbons on the substrate.

The crystalline ribbons grown on the substrates were fabricated as top-contact, bottom-gate configuration transistors. The gold source and drain electrodes were deposited with copper masks covering the selected ribbons. With this simple method, 50 nm of Au was deposited on the covered substrate; and then, the masks were removed from the ribbon surface and the ribbon devices with a length of about 20 μm were manufactured. The electrical characteristics of the devices were measured at ambient conditions. The transfer and output curves are displayed in Fig. 4a and b. The field-effect mobility (*μ*) was extracted from the saturation regime, and the mobility was calculated by the linear fitting of (*I*_DS_)^1/2^*vs. V*_G_ curves. Nearly 50 transistors have been measured and all the devices exhibited good gate modulation. According to the transfer characteristics, the mobility was probed in the range of 0.008–0.3 cm^2^ V^–1^ s^–1^ along the *b*-axis, the best hole mobility could reach 0.3 cm^2^ V^–1^ s^–1^ with a threshold voltage (*V*_T_) of –10 to –25 V and on-to-off current ratios (*I*_on_/*I*_off_) > 10^5^.

**Fig. 4:**
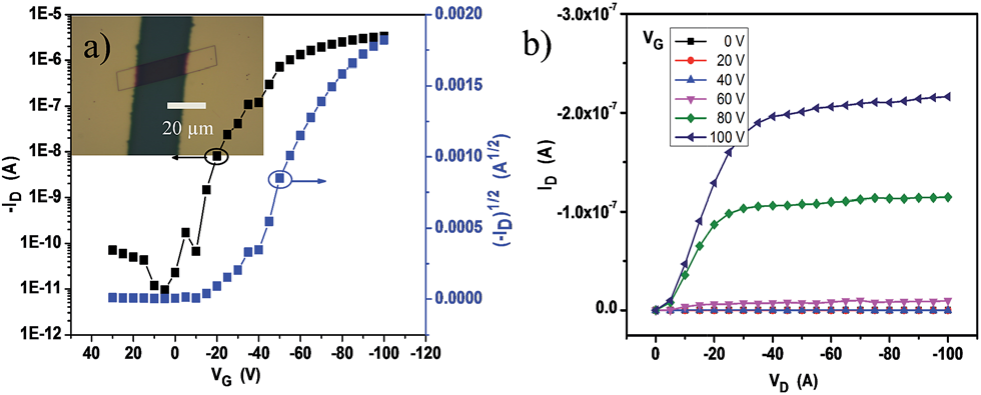
(a) Typical transfer curve and (b) output curve of the single **2BPP** ribbon transistors (inset, optical image of the device with an individual single crystal).

To understand the structure–property relationship of **2BPP**, Marcus electron transfer theory and an incoherent Brownian motion model have been employed to calculate the hole and electron mobilities (see ESI†) based on the single-crystal structure of **2BPP**.^11^ The room temperature hole and electron-diffusion mobilities were predicted to be 1.49 cm^2^ V^–1^ s^–1^ and 1.65 cm^2^ V^–1^ s^–1^ (Fig. S4 and Table S1†). The simulated hole mobility is inconsistent with the measured mobility of 0.3 cm^2^ V^–1^ s^–1^, and the π–π stacking directions show the largest transfer integrals for hole transport are 39.92 meV, which is even close to the most famous p-type semiconductor (**BTBT**) which has a transfer integral of *ca.* 60 meV. Although the material has been predicted to possess a well-balanced ambipolar property, electron transport was not apparently observed and the measured mobility was relatively low. However, we believe that with proper modification of this specific heterocyclic molecule or tuning of the device fabrication conditions, ambipolarity and good transport character could be realized.

In conclusion, we have presented the synthesis and full characterization of an unexpected “kinked” N-heteroacene (**2BPP**), in which two **BPP** units are fused together through a C=C double bond and two H-bonds. Single crystal X-ray studies have demonstrated that **2BPP** is a near coplanar molecule with close intermolecular interactions. For the double-layer structure, the face-to-face stacking mode results in an overlap between the second layer **BPP** unit and the first layer unit of the second **BPP** molecule, forming a ladder-like corrugate. The electronic structure calculations suggest the unique large heterocyclic molecule could exhibit a good intrinsic ambipolar charge transport property. Experimentally, single-crystal FETs with charge carrier mobilities of 0.3 cm^2^ V^–1^ s^–1^ and current on/off ratios of 10^5^ have been realized. Further studies on the mechanism of this unusual compound as well as the hydrogen bonding supramolecular synthons would provide more insights to design and prepare novel large conjugated heteroacenes with unique properties.

## Supplementary Material

Supplementary information

Crystal structure data
