# Supplementary material for: Fusing N-heteroacene analogues into one “kinked” molecule with slipped two-dimensional ladder-like packing
Source: Chem Sci. 2015 Nov 10;7(2):1309–13. doi: 10.1039/c5sc03604f (PMC5975899; doi:10.1039/c5sc03604f)
Supplement: Supplementary file 1 [file SC-007-C5SC03604F-s001.pdf]

Electronic Supplementary Material (ESI) for Chemical Science.
This journal is © The Royal Society of Chemistry 2015

Supporting Information

Fusing *N*-heteroacene Analogues into One "Kinked" Molecule with Slipped Two-dimensional Ladder-like Packing

Jing Zhang,[‡a] Chengyuan Wang,[‡a] Guankui Long,[a] Naoki Aratani,[b] Hiroko Yamada,[b] Qichun Zhang*[a, c]

[a]School of Materials Science and Engineering, Nanyang Technological University, Singapore, 639798, Singapore. Email: qczhang@ntu.edu.sg

[b]Graduate School of Materials Science, Nara Institute of Science and Technology, Ikoma, 630-0192, Japan

[c] Division of Chemistry and Biological Chemistry, School of Physical and Mathematical Sciences, Nanyang Technological University, Singapore, 637371, Singapore

Experimental Section

Materials

The chemical reagents and solventswere purchased and used as receivedwithout furtherpurification.1,4-bis((triisopropylsilyl)ethynyl)-2,3-diaminalphenazine was prepared according to a literature reported procedure.[1]

Synthesis

Synthesis of **2BPP**

2BPP was synthesized in the same procedure as the preparation of **THNQ**, which was obtained as dark green powder in yield of 2.3%.[2] ^{1}H NMR (300 MHz, $CDCl_3$) δ = 1.14~1.46 (84H), 7.60~7.81 (4H), 8.01~8.17 (4H), 8.55~8.71 (2H), 12.06~12.28 (2H); ^{13}C NMR (75 MHz, $CDCl_3$) δ = 11.61, 11.68, 19.08, 19.13, 98.14, 98.99, 101.45, 103.73, 108.12, 108.27, 116.63, 129.42, 129.61, 129.76, 130.17, 133.66, 134.03, 140.59, 142.37, 143.04, 143.60, 159.84.HRMS(ESI) *m/z* Calc. for $[M+H]^+$ $C_{72}H_{97}N_8Si_4O_2$: 1217.6812, found : 1217.6810.

Methods

HRMS (ESI) was recorded on a Waters Q-TofpremierTM mass spectrometer. ^{1}H NMR and ^{13}C NMR spectrawere recorded on a Bruker 300-MHz spectrometer. The investigation of UV-vis absorbance was carried out on Shimadzu UV-2501 spectrophotometer. Cyclic voltammetry was carried out with CHI 604E Electrochemical Analyzer. Glassy carbon (diameter: 1.6 mm; area 0.02 cm^2) was used as working electrode, platinum wires were used as counter electrode and reference electrode, respectively. Potentials were recorded versus Fc^+/Fc in a solution of anhydrous CH_2Cl_2 with 0.1 M tetrabutylammoniumhexafluorophosphate ($n Bu_4NPF_6$) as supporting electrolyte at a scan rate of 50 mV s^{-1}.[3,4] Fc^+/Fc was used as an external standard, and the HOMO energy level of ferrocene is -4.80 eV. Single-crystal diffraction analysis data were collected at 90 K with a BRUKER-APEX II X-ray diffractometer equipped with a large area CCD detector by using graphite monochromated Mo-Kα radiation (λ= 0.71069 Å). The structures were solved and refined by SHELXL-97 program. The hydrogen atoms were located at geometrically calculated positions and were not refined. The geometry structureswere optimized by using DFT calculations (B3LYP/6-31G*), and the frequency analysis was followed to assure that the optimized structures were stable states.To calculate the mobility, we simply take the methodology described in Shuai's review.[5,6]

Growth of the Micro-crystals and Device Fabrication: The SiO_2/Si substrate was heavily doped n-type Si wafer with a 500nm thick SiO_2 layer and a capacitance of 7.5 nF·cm^{-2}. Bare substrates were successively cleaned with pure water, piranha solution (H_2SO_4:H_2O_2 = 2:1), pure water and pure isopropanol. Treatment of Si/SiO_2 wafer with OTS used in the present study was carried out by vapour deposition method. The clean wafers were dried under vacuum at 90 °C for 0.5 h in order to eliminate the influence of the moisture. After cooling to room temperature, a little drop of OTS was placed on the wafers. Subsequently, this system was heated to 120 °C and maintained for 2 h under vacuum. Micrometer-sized single crystals of BBPP were conducted by using the drop-casting method. A toluene solution containing 2BPP (~1 mg mL^{-1}) was poured over the substrates and the solvent evaporated at room temperature. Drain and source Au electrodes (~50 nm thick) were deposited on the crystal by thermal evaporation with a copper grid as the shadow mask. X-ray diffraction (XRD) was measured on D8/max2500 with Cu Ka source (κ = 1.541 Å). *I–V* characteristics of the OFETs were recorded with a Keithley 4200 SCS and a Micromanipulator 6150 probe station in a clean and shielded box at room temperature in air.

Theoretical methodology for mobility calculations

To calculate the mobility, we simply take the methodology described in Shuai's review. The mobility is described by Einstein equation:

$$\mu = \frac{e}{k_B T} D \, (1)$$

Where the diffusion coefficient D could be estimated as

$$D = \frac{1}{2d} \frac{< x(t)^2 >}{t} \approx \frac{1}{2d} \sum_i r_i^2 W_i P_i \, (2)$$

r_i, W_i and P_i are the hopping distance, rate and probability ($P_i = W_i / \sum_i W_i$). And n is the dimension of the structure.

The hopping rate W can be described by Marcus theory in the following equation:

$$W = \frac{V^2}{\hbar}\left(\frac{\pi}{\lambda k_B T}\right)^{1/2} \exp\left(-\frac{\lambda}{4k_B T}\right) \quad (3)$$

Where V is the intermolecular electronic coupling term and λ is the reorganization energy. The electronic coupling for the hole and electron can be obtained by using Prof. Shuai's code based on Eq. 4.

$$V = \frac{H_{12} - \frac{1}{2}(H_{11} + H_{22})S_{12}}{1 - S_{12}^{\ 2}} \quad (4)$$

The H matrix elements are calculated by $H_{ij} = < \phi_i | H | \phi_j >$, where φ_i and φ_j represent the highest occupied molecular orbitals (HOMOs) for hole transport of isolated molecules in the dimer. H is the self-consistent Hamiltonian matrix of the dimer and S_{12} is the overlap integral.
The intermolecular electronic couplings V (V_h for hole transfer and V_e for electron transfer) for the fourteen pathways (shown in **Figure S4**) are calculated at DFT/PW91PW91/6-31G(d) level, the hole and electron reorganization energies of **2BPP** are calculated to be 0.1807 eV and 0.1888 eV at the DFT/B3LYP/6-31G(d) level. The electronic couplings are given in **Table S1**.

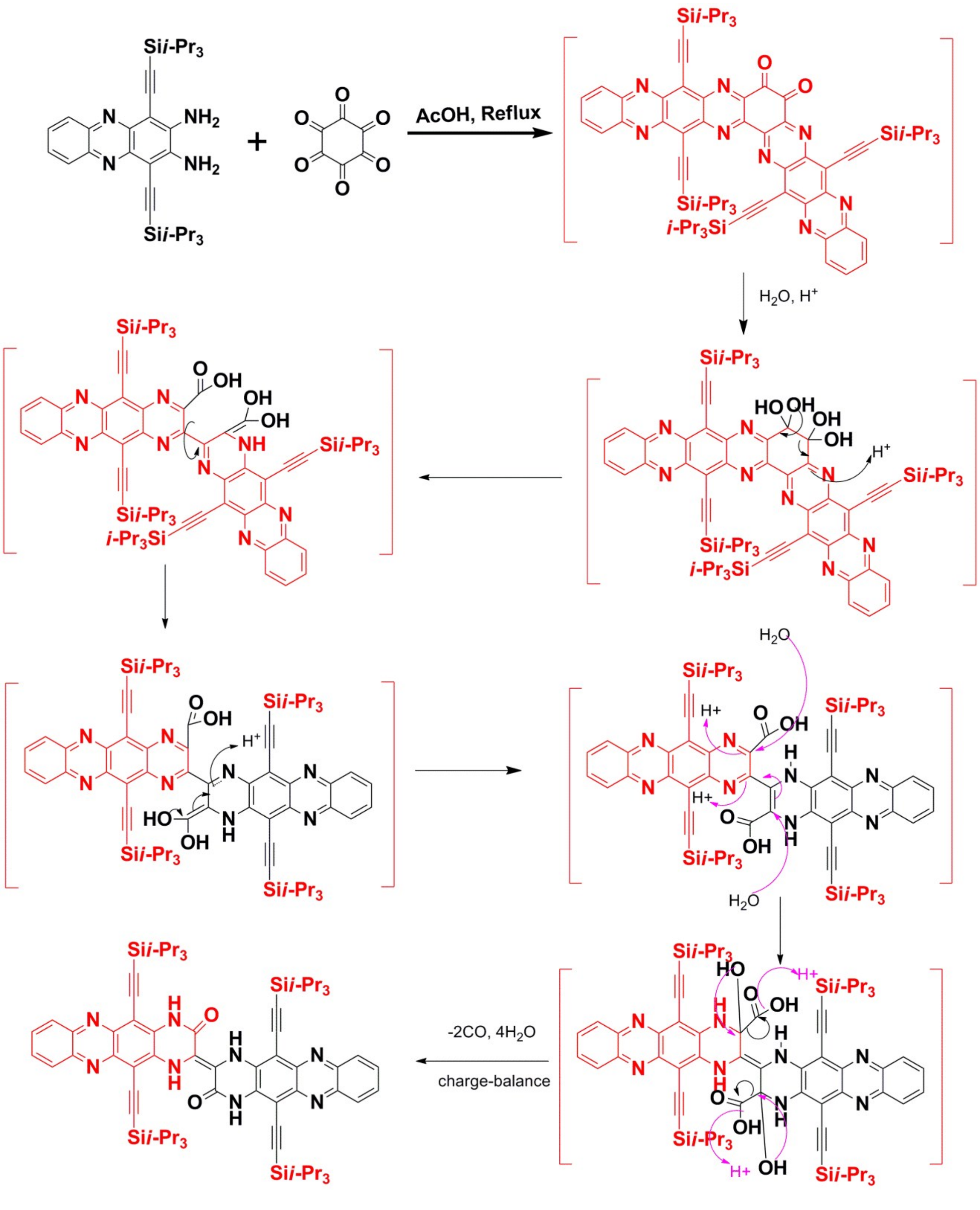

Scheme S1 the possible mechanism to form **2BPP**

Figures and Spectra

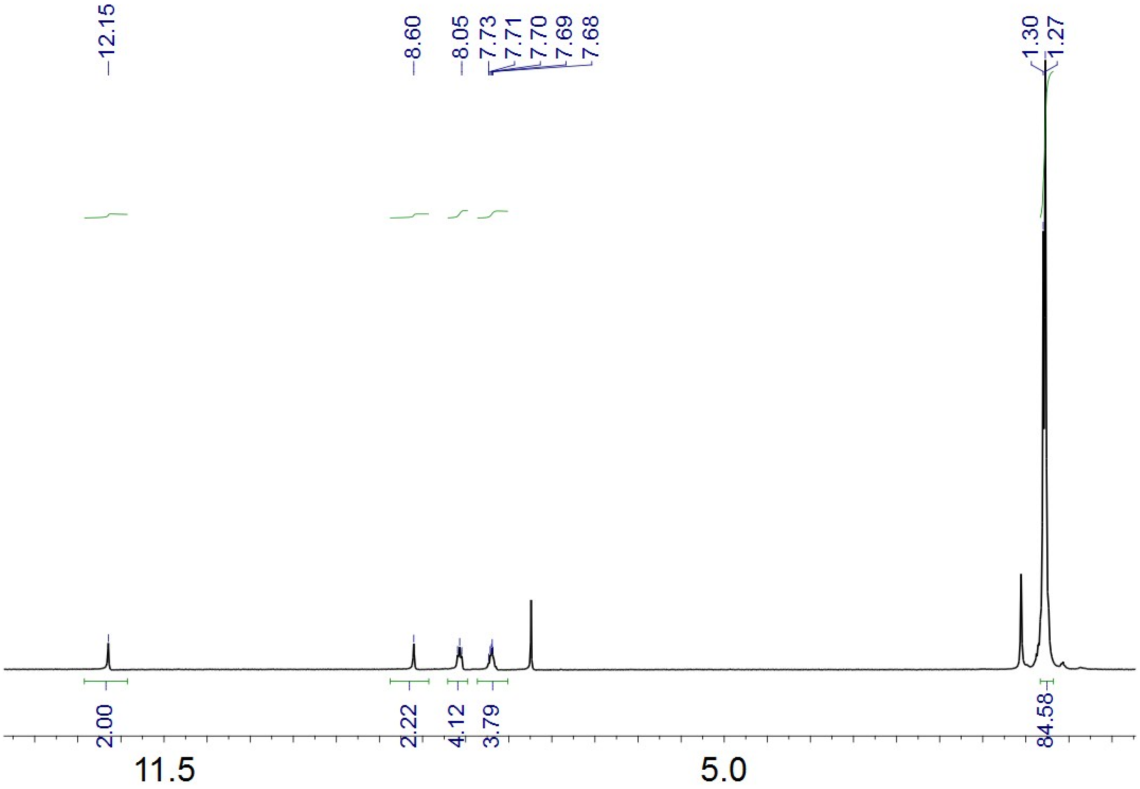

Figure S1 ^{1}H NMR spectra of 2BPP.

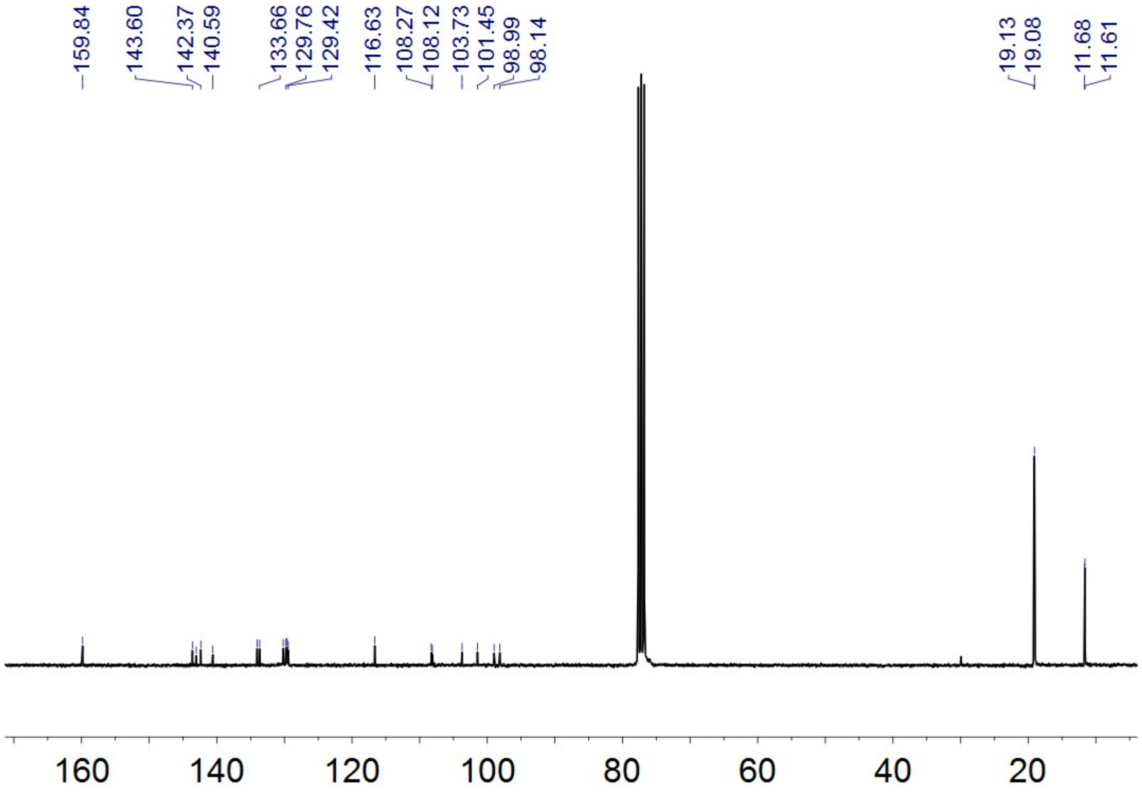

Figure S2. ^{13}C NMR spectra of 2BPP.

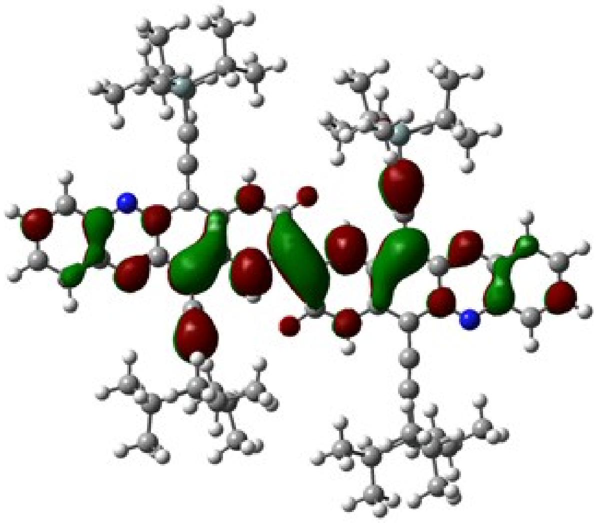

HOMO

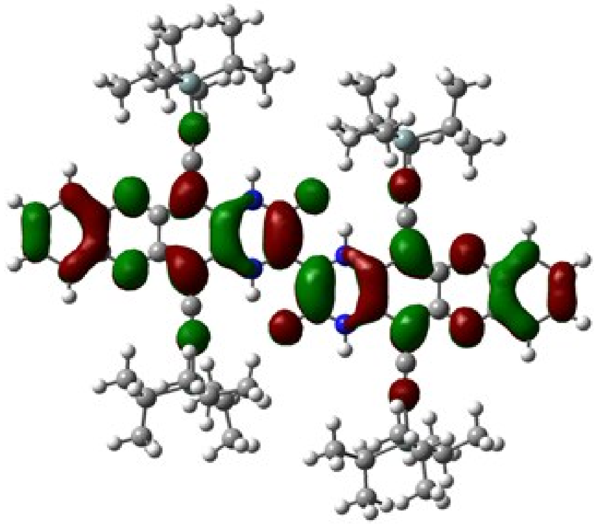

LUMO

Figure S3.The calculated frontier orbitals for the 2BPP

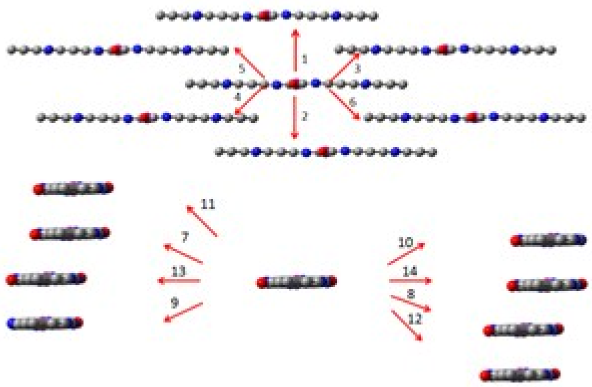

Figure S4. Hoping routes in the **2BPP** crystal (all the hydrogen atoms and TIPS were omitted for clarify).

Table S1 The electronic couplings (V) for all the hopping pathways of compound **2BPP**.

pathway	center-center/Å	V_h/meV	V_e/meV
1	7.51	-0.413	0.148
2	7.51	-0.412	0.146
3	14.65	-39.915	14.98
4	14.65	-39.915	14.977
5	17.61	4.843	-38.859
6	17.61	4.841	-38.861
7	17.27	-0.138	0
8	17.27	-0.138	0
9	18.94	0.001	0.001
10	18.94	0.001	0.001
11	21.28	0	0
12	21.28	0	0
13	23.50	0.024	-0.133
14	23.50	0.025	-0.133

References

(1)B. D. Lindner, J. U. Engelhart, O. Tverskoy, A. L. Appleton, F. Rominger, A. Peters, H.-J. Himmel, U. H. F. Bunz, *Angew. Chem. Int. Ed.***2011**, *50*, 8588-8591

(2) C. Wang, J. Zhang, G. Long, N. Aratani, H. Yamada, Y. Zhao and Q. Zhang, *Angewandte Chemie International Edition*, 2015, **54**, 6292-6296.

(3) A. Becke, *J. Chem. Phys.* **1993,** *98,* 5648-5652.

(4) C. Lee, W. Yang, R. G. Parr, *Phys. Rev. B* **1988,** *37,* 785-789.

(5) L. Wang, G. Nan, X. Yang, Q. Peng, Q. Li, Z. Shuai, Ch*em. Soc. Rev. 2*0**10, 3**9*, 4*23-434.

(6) Z. Shuai, L. Wang, Q. Li, Ad*v. Mater. 2*0**11, 2**3*, 1*145-1153.
